# The emerging role of probiotics in the management and treatment of diabetic foot ulcer: a comprehensive review

**DOI:** 10.3934/microbiol.2025027

**Published:** 2025-07-24

**Authors:** Abrar Hussain, Naheed Mojgani, Syed Muhammad Ali Shah, Nazia Kousar, Syed Abid Ali

**Affiliations:** 1 Third World Center for Science and Technology, H.E.J research Institute of Chemistry, International Center for Chemical and Biological Sciences, University of Karachi, Karachi-75270, Pakistan; 2 Biotechnology Department, Razi Vaccine and Serum Research Institute-Agriculture Research, Education and Extension Organization (AREEO), Karaj, Iran; 3 Faculty of Veterinary and Animal Sciences, University of Poonch Rawalakot, Azad Kashmir, Pakistan; 4 Faculty of Pharmacy, The University of Lahore, Lahore, Pakistan

**Keywords:** Diabetic mellitus, diabetic foot ulcers, probiotics, microbiota, dysbiosis, gut microbiome

## Abstract

Diabetic foot ulcer (DFU) is a complex complication characterized by tissue damage and neurological problems in the lower extremities. Poor wound healing intensifies the severity of DFU, which currently has a 15%–20% prevalence and thus poses a significant healthcare challenge. DFU treatment is often considered complicated due to multifaceted problems, including high cost, low stability, and prolonged healing time. Thus, there is a need to find multidisciplinary, cost-effective, and potential treatment options. In parallel, the role of skin and gut microbiota has been highlighted, influencing the progression of DFU. Probiotics, when used in sufficient amounts, confer a health benefit to the host and are found to have a promising treatment potential for DFU. Probiotics exert beneficial effects that help to improve the management and healing of DFU, following various mechanisms like controlling hyperglycemia, enhancing immune function, modulating the microbiota, and maintaining glucose homeostasis, all of which contribute to improved management and healing of DFU. Despite the potential of probiotics in DFU treatment, their precise mechanisms, optimal strains, dosages, and experimental validation remain underexplored. To fully explore the probiotic potential for DFU, extensive animal studies and clinical trials are needed. This article provides a comprehensive overview of the current status of DFU, existing treatment options, current limitations, and the growing importance of probiotic therapy. It also emphasizes the application of advanced technologies, including artificial intelligence (AI) and machine learning (ML), in advancing DFU treatment strategies.

## 1. Introduction

Diabetic foot ulcer (DFU) is a multifactorial and debilitating complication of diabetes, characterized by chronic wounds that are prone to infection and are difficult to heal [1]. Despite advancements in wound care, the global burden of DFU remains significant owing to foot amputations and substantial healthcare costs [2],[3]. Studies indicate that 19%–34% of adults with diabetes are at risk of DFU development, presenting a significant burden on healthcare systems [4]. According to the International Diabetes Federation, approximately 40–60 million individuals globally are affected by DFU [5]. DFU indicates more severe microvascular complications and poor health status [5],[6]. Chen et al. (2023) reported in a meta-analysis comprising 125,000 patients from 16 countries that there is a 13.1%, 49.1%, and 76.9% mortality rate at 1, 5, and 10 years following an incident of DFU, with cardiovascular disease and infection being the leading causes of death [7]. Conventional treatments of DFUs primarily rely on surgical debridement, antibiotic therapy, advanced wound dressings, and, in severe cases, amputations [8],[9]. The treatment of DFU is considered challenging due to the multiple phases, causes, and varying degrees of severity that exist among patients with diabetes mellitus [8],[10]. Additionally, most of the treatments are expensive and have limitations. For instance, wound dressings often fail to adequately absorb exudates, and while antibiotics may reduce microbial load, they are generally ineffective in promoting the long-term healing of DFU [11].

Due to limitations in current treatment options, researchers are seeking novel and natural ways to manage and treat DFU. In the recent decade, the potential of probiotics has been extensively explored as a novel therapeutic strategy for DFU [12]. Probiotics could be utilized as an adjunct therapy for diabetes mellitus and DFU. The role of the gut microbiome in the physiology and disease treatment of an individual is well known [13],[14]. It is now clear that changing the gut microbiota can change the behaviors, mood, and even cognitive functions of an individual [15], highlighting the role of desirable microbes, including probiotics, in restoring the gut microbiota and providing a potential option for targeting gut dysbiosis complications. Besides gut restoration, probiotics have numerous health benefits, which make them an important target for disease treatment [16],[17].

Considering the increasing global burden of DFUs and the limitations of current treatment modalities, there is growing interest in alternative and adjunctive therapies. Probiotics, having the potential to modulate gut microbial communities, improve immunity, exhibit anti-inflammatory and antimicrobial properties, and have promising potential in various metabolic and inflammatory diseases, including diabetes mellitus (DM). However, their role in DFUs remains underexplored. Hence, this article aims to explore the emerging therapeutic potential of probiotics in the management and treatment of DFU. This review also highlights the basics of probiotics and DFUs, while emphasis is given to exploring their associations. A special focus is placed on understanding the mechanisms by which probiotics may influence wound healing, reduce pathogenic microbial burden, and enhance immune modulation in the context of DFUs. Through a critical analysis of existing studies and identification of current research gaps, this review highlights the potential of probiotics as a novel, cost-effective, and adjunctive strategy in DFU management.

## 2. Probiotics and their properties

At the beginning of the 20^th^ century, the idea of probiotics was developed, relying on the idea that changing gut microbiota with friendly bacteria could enhance the life span of an individual [18]. Over time, researchers took an interest in this field and developed its different aspects, like its proper definition, new strains identification, selection criteria, and commercialization. In 2002, the WHO and FAO defined probiotics; the term was updated in 2013 by the International Scientific Association for Probiotics and Prebiotics (ISAPP), who proposed a consensus statement. According to these statements, probiotics are “live microorganisms that, when administered in adequate amounts, confer a health benefit to the host” [19]. The most common probiotics belong to the group of lactic acid bacteria (LAB), dominated by lactobacilli and *Bifidobacterium*, followed by *Enterococcus* [20]. Usually, the Generally Recognized As Safe (GRAS) and Qualified Presumptions of Safety (QPS) status microorganisms are used as probiotics, while occasionally strains from other genera, like *Enterococcus*, are also used [21],[22]. Currently, the field is growing rapidly and covers a wide spectrum of applications.

**Figure 1.**
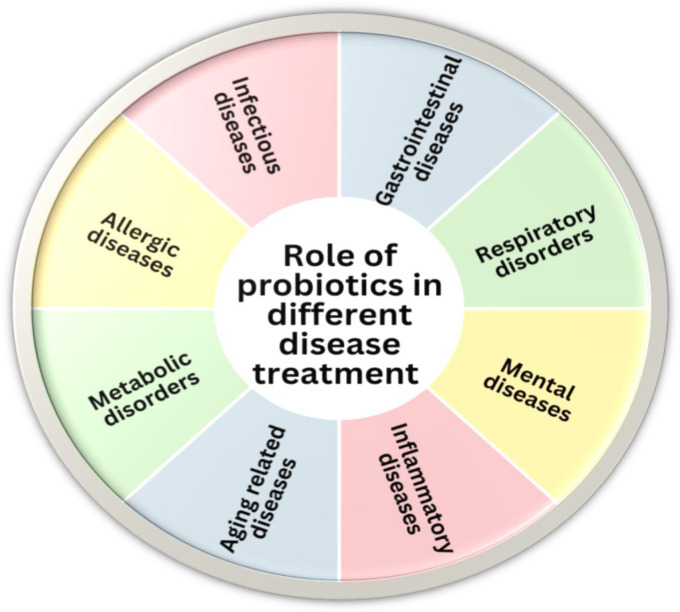
Overview of the preventive and therapeutic roles of probiotics across different diseases.

Probiotics are strain-dependent phenomena, meaning that different strains, including those from less common sources, can be utilized for their selective beneficial effects. For characterizing probiotics, certain selection criteria exist, like safe nature, absence of virulence and antibiotic resistance, survival in acidic environments, pathogen killing, and production of bacteriocins, which are followed during strain selection [20]. Probiotics have characteristics that make them promising therapeutic agents and important biotechnological entities. Survivability, adherence, immunomodulation, tolerance, aggregation, and postbiotic production are considered unique properties that contribute to their therapeutic potential [15],[23]. The intrinsic characteristics of probiotic strains, along with their beneficial functional properties, make them a subject of growing interest among researchers [24]. Currently, probiotics have wide applications in food, pharmaceuticals, cosmetics, and other biotechnological industries. Likewise, the health profile of probiotics is also wide, and they are currently used for the prevention and treatment of different metabolic, respiratory, infectious, digestive, and neurological disorders [25]–[28]. The anti-aging, anti-inflammatory, antioxidant, and anti-pathogenic applications of probiotics have been widely investigated, indicating their health benefits and other therapeutic potentials [15],[29],[30]. The different diseases that are treated with probiotics are summarized in Figure 1.

### 2.1. Overview of diabetes mellitus

Diabetes mellitus (DM) is a metabolic disease characterized by inadequate regulation of blood glucose levels, either due to the insufficient secretion of insulin by pancreatic β cells or by the insensitivity of insulin receptors to oxidize blood glucose [31]. DM encompasses various subtypes, including type 1, type 2, gestational diabetes, monogenic diabetes, and other secondary diabetes [32],[33]. Among these subtypes, type 1 and type 2 are the most well-known and important subtypes, each with distinct pathophysiological mechanisms, clinical presentations, and therapeutic approaches; yet, both types are associated with hyperglycemia. According to reports, DM could affect various organs and lead to different complications such as strokes, kidney failure, vision loss, and the amputation of lower limbs [34].

The prevalence of DM is alarming in the modern era. According to the World Health Organization, in 2014, approximately 8.5% of individuals aged 18 and above were impacted by diabetes [35]. In 2019, diabetes accounted for 1.5 million deaths, of which 48% occurred below the age of 70 (WHO). In 2021, diabetes was the reason for 1.6 million deaths, and 47% of all these deaths occurred before the age of 70 [36] (WHO, 14 November 2024). The treatment of DM is considered complex and requires different interventions for its management. Lifelong treatment is often required in order to avoid unwanted complications [31]. Although controlling blood glucose levels is highly critical, excessively aggressive management might result in hypoglycemia, which may have adverse or fatal consequences [33]. The American Diabetes Association (ADA) advises routine blood pressure checks for individuals with diabetes. The medications recommended for treating hypertensive diabetics include enzyme inhibitors, receptor blockers, and diuretics [32],[37],[38]. Due to the higher prevalence of DM, healthcare providers have been searching for more efficient, effective, and safe treatment options [37].

## 3. Diabetic foot ulcer

Diabetic foot ulcer (DFU) is the most common, serious, and exponentially growing public health problem faced by diabetic patients [1],[39]. It is a complex and multifactorial clinical issue that impacts many diabetic patients, who experience ulceration and infection [40]. It is characterized by an open wound, typically located on the plantar surface of the foot, which is often associated with hemorrhagic tissue damage [5],[41]. The wound is initially superficial but progresses with time, leading to necrosis of the affected area and spreading infection deeper and forward toward the upper portion of the limb. It is often accompanied by neuropathy and/or peripheral artery disease that disrupts the foot epidermis and dermis, breaches the skin barrier, and exposes sterile structures that ultimately result in full-thickness lesions [42]. According to the International Working Group on the Diabetic Foot (IWGDF) guidelines, DFU is defined as a condition resulting from current or previous diabetes, characterized by symptoms such as ulceration, skin chapping, or destruction of foot tissues [9],[42],[43].

**Figure 2.**
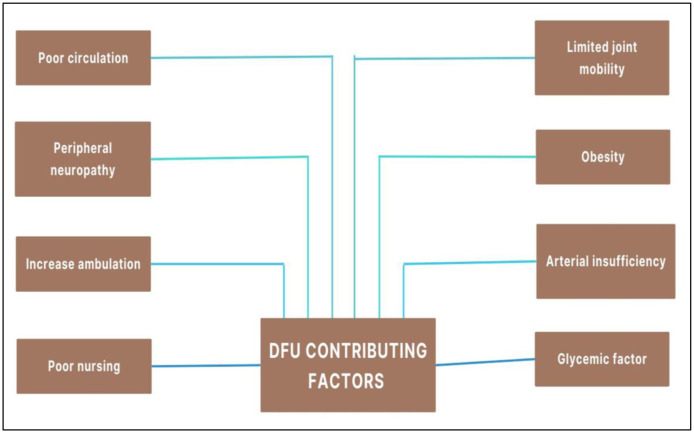
Different factors contributing to the occurrence, prevalence, and consequences of diabetic foot ulcers.

In the developed world, over 60% of non-traumatic amputations are associated with DFU, contributing to higher hospitalization rates, increased mortality, and diminished quality of life [42],[44]. Limb amputations could impose significant strain on the economic and healthcare resources of the diabetic patient [45]. It occurs due to the infection of bacteria, followed by their colonization at the site of the wound. Initially, a hemorrhagic wound occurs. Then, a callus forms, which tears off, exposing the tissue to pathogenic invasion [46]. As reported, the two main causes of DFU are damage to any of the peripheral nerves (including the superficial fibular nerve, deep fibular nerve, tibial nerve, sural nerve, and saphenous nerve) and ischemia of the foot extremities [47]. The person does not feel any inflammatory responses, and the tissue damage continues, leading to DFU [48]. Factors contributing to DFU are illustrated in Figure 2.

Often, individuals with type 1 or type 2 DM experience injuries requiring complex and prolonged treatments and are known to have DFU [39]. The presence of many proliferating macrophages at the site of injury for a long time can exacerbate the damage, leading to the development of a diabetic wound [5],[45]. Moreover, patients with diabetic feet are at high risk of bacterial infections. Impaired microvascular circulation at the site of injury in DFU patients restricts the entry of phagocytes, leading to increased infection risks [49]. The common pathogens that contribute to the DFU progression are Gram-positive aerobes (e.g., *Staphylococcus aureus*, *Staphylococcus epidermidis*), Gram-positive anaerobes (i.e., *Propionibacterium spp*., *Peptostreptococcus spp*., *Peptococcus s*pp., and *Streptococcus spp*.), Gram-negative aerobes (i.e., *Pseudomonas aeruginosa* and *Acinetobacter spp*.,), and Gram-negative anaerobes (*Proteus mirabilis* and *Bacteroides spp*.) [50]–[55]. Despite higher oxygen levels in the wounds, several anaerobes, such as *Finegoldia*, *Prevotella, Peptoniphilus*, and *Anaerococcu*s, pose major threats [56]. Fungi, mainly *Candida spp*., have also been detected as microbial flora in DFU patients, contributing to wound complexity and slowing the healing process [51],[56]. Reports have shown that low-income countries exhibit a higher prevalence of Gram-negative pathogens, with *Pseudomonas aeruginosa* being the most commonly observed bacteria in DFU, in contrast to the developed countries, where *Staphylococcus aureus* is the dominant species. This discrepancy is probably due to lifestyle, environmental conditions, and hygienic practices [57].

According to the International Diabetes Federation (IDF), the prevalence of diabetes will increase by 10.2% by the year 2030, with the number of affected individuals increasing from 463 million in 2019 to 578 million [3],[58]. The risk that a diabetic patient may develop DFU in their lifetime ranges from 15% to 34% [59]. Studies have revealed that about 20% of the legs of diabetic patients are amputated because of DFU [60]. In Zhang et al. (2017), it was reported that the prevalence of DFU in diabetic patients over the past 3 decades is 6.3%, with the highest value of 13.0% being found in North America and the lowest in Europe, at about 5.1% [61]. The mortality rate of DFU patients is extremely high and observed to be 50% within 5 years of occurrence, with patients suffering from cardiovascular diseases and infections along with diabetes [62],[63].

**Figure 3.**
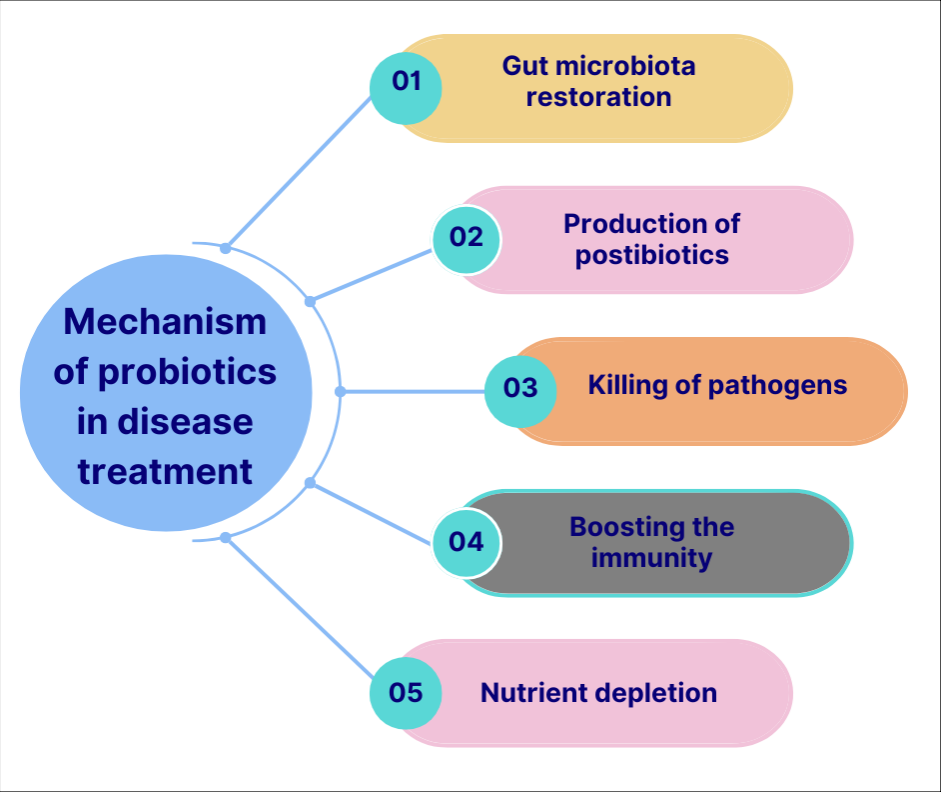
Mechanisms by which probiotics impart beneficial health effects on the host.

Microorganisms are found in all environments, with varying species prevalence, and commonly being the causative agents of many diseases [64]. Besides the general devotion to being pathogenic, these microbes are also harnessed for their biotechnological applications. An important group of microorganisms, termed probiotics, has been highly investigated for their ability to cure diseases and promote life [18]. The disease-treating potential of probiotics is vast, although they do not have a dose profile like a drug; they are still used as medicine for different disease treatments. Cognitive functions, allergies, digestive disorders, aging, respiratory syndromes, and immunity-related diseases are a few common diseases that are prevented or treated with probiotics [65],[66]. The health-promoting effects of probiotics are mediated through various mechanisms, many of which are strain-specific. However, several core functional pathways are commonly observed across different probiotics. These include the production of antimicrobial substances, modulation of the host immune responses, attenuation of pro-inflammatory signaling pathways, and scavenging of reactive oxygen species through antioxidant enzyme activity. These multifaceted actions reinforce the therapeutic value of probiotics in maintaining host homeostasis and preventing or mitigating various pathological conditions [15],[67]. As shown in Figure 3, probiotics bestow health benefits and provide protection against several diseases via different mechanisms of action.

### 3.1. Current treatment options for DFU

As mentioned, DFU prevalence is increasing due to multiple contributing factors. These factors are variable and can be controlled at different checkpoints. To comprehensively understand the potential role of probiotics in the management of DFUs, it is essential to first examine the current therapeutic strategies. Reviewing existing treatment modalities provides foundational insight into their mechanisms, effectiveness, and inherent limitations. Furthermore, a critical evaluation of these approaches highlights the need for innovative interventions aimed at addressing unresolved challenges and enhancing clinical outcomes. The current treatment options for DFU are shown below.

#### 3.1.1. Debridement

The first approach adopted whenever DFU is presented to the physician is debridement. Debridement is regarded as the removal of dead or necrotic tissue and the elimination of any foreign objects present at the site of the wound. Debridement may be done using surgical, autolytic, enzymatic, biologic, or mechanical approaches [59],[68]. Debridement involves the removal of necrotic tissue, which, although it may cause some damage to surrounding healthy tissue, ultimately accelerates the healing process [69]. This is because the old necrotic wound converts into an acute wound, resulting in more rapid granuloma formation and epithelization processes, which contribute to the healing process [70].

#### 3.1.2. Glycemic control

Glycemic control in the normal range has proven to be a beneficial strategy to manage DFU in patients suffering from diabetes mellitus. Many studies have proven the positive relationship between glycemic control and DFU patients [71]. Glycated hemoglobin (HbA1c) can be used as the determinant of glycemic control in DFU patients [72]. Lane et al. (2020) reported that patients with HbA1C levels ≥8% and fasting glucose levels ≥126 mg/dL are more likely to have lower extremity amputation than controlled glycemic control patients with DFUs [73]. Hence, glycemic control can be used to decrease amputation incidence in DFU patients with diabetes [74].

#### 3.1.3. Offloading

Offloading lowers the pressure, stress, and ambulatory activities of the leg suffering from DFU. Offloading can decrease the chances of amputation of the lower limb and is considered one of the foundational strategies for the management of wounds in DFU patients [75]. Various offloading strategies can be employed for DFU management, including offloading devices, surgical offloading, and general offloading techniques such as placing a foam pad under an ulcerated foot [76]. The total contact cast (TCC) and knee-high devices are the most commonly used and reliable offloading devices, which are recommended by physicians for managing DFUs [77],[78]. A study observed that 96% of DFUs are healed in less than one month by surgical offloading, while 68% of ulcers healed within 3 months by standard care, highlighting the effectiveness of surgical offloading [79].

#### 3.1.4. Infection control

Infection is the invasion of bacteria at the site of DFU. Infection at the wound site can cause tissue degeneration due to toxins and reactions by microorganisms, consequently leading to necrosis and ultimately gangrene. Infection is the cause of 80% of amputations in patients suffering from DFUs [12]. Kee et al. (2019) observed that 14.8% of the DFUs patients healed within one to six months, while 27.6% of them took more than 6 months to be healed while following proper wound care and offloading [80],[81]. Topical and systemic antimicrobial therapy is also recommended for infection control if the patient is suffering from palmar DFUs [82].

#### 3.1.5. Surgery

Surgical interventions are the only option left when the patient does not respond to any antimicrobial treatment. The physical removal of tissues, from debridement to amputation, comes under the roof of surgical innervation [83], although revascularization is considered crucial before debridement of dry gangrene and the reconstruction of diabetic foot ulcers. Revascularization aims to restore blood flow to the ischemic tissues, providing the necessary oxygen and nutrients for healing. Without revascularization, debridement and reconstruction can be ineffective or even harmful, leading to increased risk of amputation and other complications. Revascularization can be achieved through various methods, such as angioplasty or bypass surgery, depending on the location and extent of the vascular blockage. While revascularization is crucial for healing, debridement is often necessary to remove the dead tissue and reduce the bacterial load. In dry gangrene, debridement can be delayed until blood flow is improved. However, in cases of wet gangrene, where infection is present, debridement may be necessary before or concurrently with revascularization to control the infection.

Likewise, in the management of DFU, particularly those caused by peripheral artery disease (PAD), sufficient blood supply is often lacking to promote healing. This is because the blockage or narrowing of arteries in the leg and foot restricts the delivery of oxygen and nutrients to the wound site, hindering tissue repair. Revascularization plays a critical role by enhancing the blood flow to the ulcer, delivering essential oxygen and nutrients necessary for tissue regeneration and wound closure. This process produces a more favorable environment for any succeeding surgical procedures, such as debridement or reconstruction, by ensuring adequate blood supply to the affected area [164]–[166].

Surgeries are reported to achieve 52% healing rates in patients suffering from DFUs within 12 months of the procedure. However, some studies show higher healing rates, while others indicate that a significant percentage of patients may require additional surgeries or even amputations [84]. In the case of neuropathic DFUs, the chance of reoccurrence of nerve compression reduces to 5% after surgery. Studies revealed that this is due to the surgery relieving pressure on the nerves, allowing for improved nerve function and blood circulation, and thus lowering the likelihood of ulcer recurrence [62]. A new technique for the fixture and closure of DFU wounds using podoplastic procedures is also used by physicians and has shown good results [85].

### 3.2. Role of gut and skin microbiota in DM and DFU

Bacteria, fungi, and algae, generally known as microorganisms, constitute approximately 159,000 species and are found in all environments [64]. In humans, a portion (500–1000 different types of species) of this ocean of microbes is found in the gut, which is collectively called the gut microbiota. Likewise, a large number of species are also found in or on the skin, being called the skin microbiota. The presence of these microbes in the body can affect its overall physiological functions and contribute to reshaping them [86],[87]. It is also understood that the dietary habits of an individual can impact the gut microbiota and potentially affect their metabolism [88]. Alterations in the bacterial composition of the gut microbiota, a condition referred to as dysbiosis, have been found to contribute to inflammation, which is believed to be the initial stage of disrupted gut equilibrium in individuals with diabetes [89]. The composition of the gut microbiota is influenced by metabolic health and diet, showing differences between lean and obese individuals. The onset of obesity and diabetes is increasingly associated with low-grade inflammation, which is believed to result from metabolic disturbances triggered by interactions between the immune system and gut microbiota-derived products [90]. In a previous study, transplanting gut bacteria from lean donors to insulin-resistant recipients led to desirable metabolic effects [91].

Given the numerous treatment approaches that target the microbiota, a reciprocal relationship between diabetes and the gut microbiota becomes apparent. As shown in a vast number of published reports, the gut microbiota plays an important role in different functions, including amino acid synthesis, metabolite production, and nutrient absorption, affecting both healthy and disease states of the host [92]. A significant number of bacterial species within the gut microbiota are known to provide protection against metabolic diseases such as metabolic syndrome, obesity, and type 2 diabetes [93]. Le Chatelier and his colleagues (2013) reported that obese individuals with lower microbial diversity and lower metagenomic compositions are more susceptible to obesity, adiposity, insulin resistance, and inflammation [94].

In DFU, not only the gut microbiota but also the bacterial interactions occurring on the surface of the skin play a crucial role in the pathophysiology and can influence the rate of wound healing [95]. Human skin harbors approximately 1200 distinct bacterial species, among which *Propionibacterium*, *Staphylococcus*, and *Corynebacterium* are the most prevalent taxa [96]. Furthermore, evidence indicates the presence of other bacterial species like *Cutibacterium*, *Micrococcus*, *Bacillus*, *Roseomonas*, and *Paenibacillus*, which play a role in the skin microbiome [97],[98]. The microbiota residing on our skin directly influences the skin health and disease status by cooperating with various cells that are involved in the wound healing process [95]. Specifically, commensal microbiota interacts with those skin cells that are responsible for repairing wounds, thereby promoting the generation of the skin barrier [95],[99]. Among the commensal skin microbes, *S. epidermidis* has been shown to boost innate immunity by regulating the activity of gamma delta T cells and inducing the expression of perforin-2 [100].

### 3.3. Probiotics and diabetic foot ulcer: management and treatment

**Figure 4.**
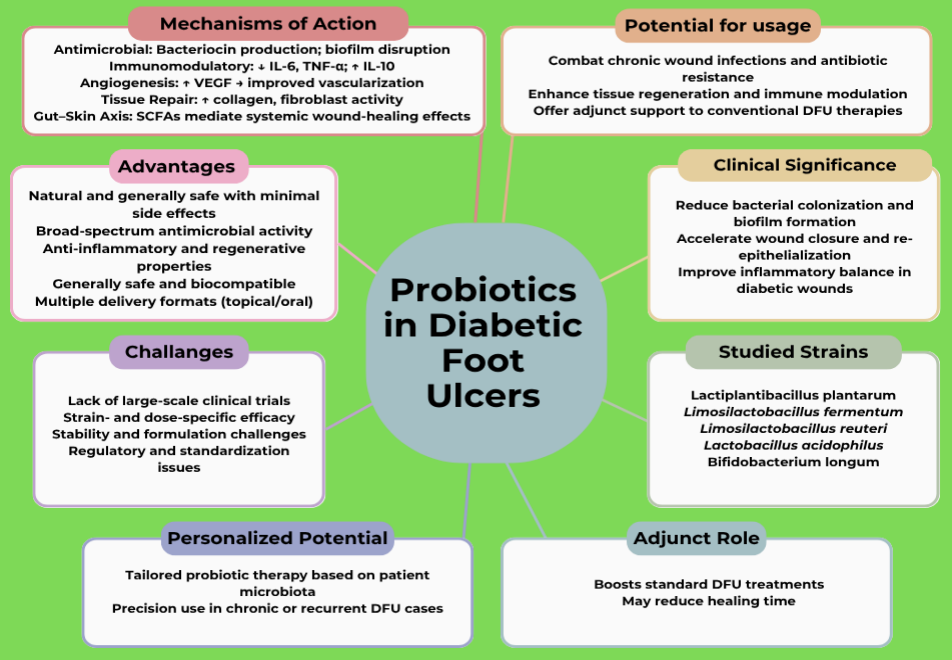
Summary of the different aspects of probiotics in the context of diabetic foot ulcers (DFUs), highlighting the rationale for their use, commonly studied probiotic strains, therapeutic advantages, and current challenges in clinical application.

Probiotics, namely viable bacteria belonging to the dominant group of LAB, have been recognized as beneficial to host health for decades [101]. Probiotics have proven to be beneficial in various diseases, including allergies, gastrointestinal disturbances, cancers, and cardiovascular disorders, and have antioxidant, anti-inflammatory, and immunomodulatory properties [20],[102]. These beneficial bacteria reportedly contribute to treating DFUs and are believed to have oxidative stress-releasing and inflammation-inhibitory effects, as diabetic patients have reduced anti-inflammatory capability [103]. Many probiotic species and their strains have anti-diabetic properties, including strains from lactobacilli, *Bifidobacterium*, and enterococci [104]. These probiotics are believed to be therapeutically beneficial both in the case of ingestion and topical application in DFU [12]. Probiotic bacteria have demonstrated a potential role in facilitating the treatment of dermatological conditions, such as wounds, venous or arterial ulcers, atopic dermatitis, acne vulgaris, and DFUs. [14],[105]. The aforementioned probiotic properties strongly support the idea that they can be used as an alternative or an adjunct therapeutic option to the conventional treatment approaches used for treating DFUs [103],[106]. The multifaceted role of probiotics in the different aspects of DFUs is summarized in Figure 4.

Probiotics have gained immense clinical interest for treating DFUs due to their remarkable wound-healing capabilities. In various animal-based studies, the application of different strains of probiotics has proved effective in controlling various DFU risk factors [104],[106],[107]. Evidence also supports the wound-healing capabilities of probiotics in cutaneous wound healing in animals [108]. Studies conducted by González et al. (2018) described that the lyophilized conditioned media *Lactobacillus acidophilus* can be helpful in inhibiting *Pseudomonas sp*. and *Enterobacter sp*. of the DFU patient's wounds [109]. Their study showed that a dosage of 400 and 800 mg/mL of *L. acidophilus* is effective for inhibiting *Pseudomonas sp*. and *Enterobacter sp*., respectively. According to their results, probiotics appeared to be effective in their antibiotic activity, similar to that of clindamycin. *L*. *acidophilus* was hence identified as an alternative approach for the treatment of patients suffering from DFU against bacteria sensitive to cefotaxime and clindamycin [109].

Probiotics not only strengthen the epithelialization of ulcerated wounds but also take part in immunomodulation, thus promoting wound healing [110]. Recently, Hou et al. (2022) reported that probiotics and prebiotics, when used in combination (synbiotics), show astonishing antimicrobial properties [111]. Probiotics like *Lacticaseibacillus casei* embedded in chitosan can be used for wounds because of their optimal delivery to the site and excellent antibacterial, antifungal, antiviral, and therapeutic effects [14],[112].

### 3.4. Mechanism of action of probiotics in diabetic foot ulcer

**Figure 5.**
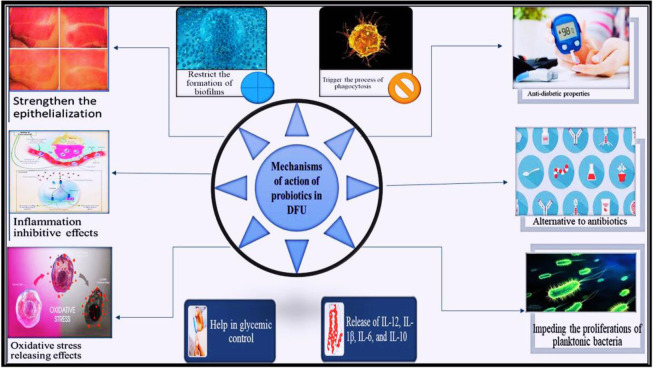
Role of probiotics in the management and treatment of diabetic foot ulcers. Probiotics enhance epithelial strength, inhibit inflammation, provide oxidative stress-lowering potential, inhibit biofilm formation, and release interleukin-6, 10, and IL-12.

Even though the exact mechanism of probiotics in wound healing is not yet clarified, there are proposed mechanisms to explain how probiotics can help in DFU management and treatment [103],[113]. Gudadappanavar et al. (2017) reported that probiotics could enhance the recovery process by impeding the proliferation of planktonic Gram-positive and Gram-negative bacteria [114]. Probiotics can inhibit biofilm formation by releasing molecules that disrupt pathogen quorum sensing and prevent adhesion to epithelial tissues [115],[116]. Figure 5 illustrates the general mechanism of action of probiotics in DFU.

A key factor that hinders the healing process of a wound is the abundance of microorganisms in the wound. The greater the number of microorganisms, the more difficult it becomes to facilitate healing [117]. Furthermore, tissue injury could alter microbiota, which allows excessive proliferation of pathogenic species and exacerbates damage. A potential characteristic of probiotics is their ability to compete with pathogens for nutrients and adhesion sites on the host's mucous membrane [26]. Their antagonistic actions are mediated by the production of lactic acid and bacteriocins that provide protection against the colonization of pathogenic species, hence preventing bacterial infections at the wound [118]. Probiotics could trigger the process of phagocytosis by inhibiting their growth and adhesion, resulting in a reduction of bacterial load in the wound [14]. Likewise, probiotic bacteria exert antimicrobial actions by regulating the production of antimicrobial peptides by the skin cells, mediating a balance in the composition of the skin microbiota and enhancing skin integrity [108].

Probiotics can help in the glycemic control of diabetic patients suffering from DFUs. It is thought that probiotics can be helpful in reducing the amputations of DFU patients [119],[120]. A mixture of probiotics containing lactobacilli (acidophilus, casei, and fermentum) and *Bifidobacterium bifidum* led to a significant decrease in HbA1c levels compared to the control group. The experimental group showed better recovery than the control group, and a remarkable decrease in DFU was observed [121]. In Ruan et al. (2015), it was observed that probiotics can lower fasting blood glucose levels, which reflects the role of probiotics on glycemic control [122].

In patients suffering from type 2 diabetes, the inflammatory responses are more active and intense than in a normal person, which interferes with and delays the healing process [123]. The high glycemic concentration and excessive release of IL-6, along with tumor necrosis factor, delay the mechanism of wound healing [124]. The two fungi-based probiotics *Kluyveromyces marxianus* and *Saccharomyces boulardii* have anti-inflammatory responses, as they prompt the release of anti-inflammatory factors, including different interleukins (i.e., IL-12, IL-1β, IL-6, and IL-10), following different mechanisms [125]. *K. marxianus* enhances Treg cells and secretes IL-10, while *S. boulardii* triggers the Th1-type response while producing IFN-γ [125]. *Latilactobacillus sakei* also decreases the release of different substances (chemokines, cytokines, etc.) that are generated from epithelial cells, reducing inflammation [126]. All these suggest that specific strains of probiotics can be used to decrease the inflammation of DFU patients, thereby relieving pain and promoting the healing process in diabetic patients. Yang et al. (2023) conducted a multicenter study for the management of DFU using topical probiotics in a soy bean concentrate and found that 83% of patients were completely healed at 16 weeks, while 72% were completely healed at 12 weeks. This study describes a new therapeutic and promising potential of probiotics for the management and treatment of DFUs [167]. Table 1 summarizes different studies using animal models and human subjects while using probiotics for the treatment of diabetes mellitus and DFU.

**Table 1.** In vitro, in vivo, and clinical trial studies evaluating the role of probiotics in cutaneous wound healing and DFU management.

Probiotic source	Application mode and duration of study	Study description	Outcomes	References
*Lactiplantibacillus plantarum*	Topical	Pilot study III 14 diabetic and 20 non-diabetic patients with chronically infected leg ulcers	Reduced bacterial loads, increased immune cells, enhanced healing, and modified inflammatory production	[127]
*L. acidophilus, B. bifidum, Lacticaseibacillus casei, Limosilactobacillus fermentum*	1 probiotic capsule daily for 12 weeks	45–85 years of age with type 2 diabetes	Decreased FBS, improved lipid profile, and inflammatory biomarkers	[128],[129]
*Lactiplantibacillus plantarum, Levilactobacillus brevis*	Topical application for 21 days	Male Wistar rats with excisional cutaneous wounds	Significant reduction in local inflammation, increase in fibroblast cells, and accelerated wound healing	[130]
*Lacticaseibacillus casei* and its exopolysaccharide	Topical application for 14 days	DW-induced male Wistar diabetic rats	Enhanced wound contractions	[131]
*Limosilactobacillus fermentum*, *Lacticaseibacillus casei, L. acidophilu*s, *B. bifidum*	Oral supplement	Randomized double-blind placebo-controlled trials in 60 subjects aged 40–85 years with grade 3 DFU	Reduced ulcer size, downregulation of blood glucose, cholesterol, high-sensitivity reactive proteins, plasma, nitric oxide levels	[121]
*L. acidophilus*	In vitro studies	Turbidimetric method to analyze the effects of three different doses of LP against DFU bacterial isolates	Probiotics at 40, 400, and 800 mg/mL inhibited 3%, 34%, and 40% of *P. aeruginosa spp*. respectively	[109]
*Limosilactobacillus reuteri* CXCL12 expressing *Limosilactobacillus reuteri or L. lactis*	Topical application once daily for 14 days	Wounds induced in C57BL/6 mice, induction of hyperglycemia, and 0.2, 0.6, and 1 µg applied to wounds	Accelerated wound closure, reduced skin perfusion	[132]
*Lactiplantibacillus plantarum* TWK10 fermented soy milk	Topical application for 14 days	36 nine-week-old diabetic Wistar rats, diabetic-induced and wound-inflicted, with ethanol extract of TWK10-fermented soymilk and Vaseline applied	Enhances wound healing by reducing inflammation and promoting cell growth, collagen deposition, and angiogenesis	[133]
*Limosilactobacillus reuteri* ATCC 11284 extracts	Local injection	Cell culture (healthy mice gingival tissues) 8-week-old female C57BL/6 mice injected with 50 ug/mL of extract	Promotes oral wound healing by the P13K/AKT/beta catenin/TGF beta 1 pathway	[134]
*Limosilactobacillus reuteri* DSM 17938	With Eucerin ointment applied for 15 days	45 Sprague-Dawley male rats, with excision wounds made on the dorsal area of the rats	Ointment was able to accelerate the wound healing process, increase collagen deposition, and reduce the inflammation rate	[135]
*S. thermophilus*	Topical	18 albino rats treated with 10^10^ to 10^11^ CFU/mL of *S. thermophilus*	Decreased oxidative stress and improved healing process	[136]
*Lactiplantibacillus plantarum*	Topical gel twice a day for 14 days	40 mature Sprague-Dawley male rats, induced diabetes and second-degree burn wounds created	Promoted wound healing by enhancing collagen synthesis, increasing fibroblast activity and TGF-β levels, and reducing the infection risk	[137]
*Lacticaseibacillus casei* CCFM419	Orally given to mice	Type 2 diabetes induced in male mice C57BL/6J	Decreases in PBG and FBG levels and an increase in SCFAs, increase in GLP-1 and IL-6	[105]
*L. acidophilus* NCIMB 43030*, Lactiplantibacillus plantarum* NCIMB 43029*, S. thermophiles* NCIMB 30438	Topical application for 24 days	83-year-old woman with history of DM	Inhibited *Proteus mirabilis* and *Klebsiella pneumoniae* at the site of injury, completely healed the wound	[139]
*Lactiplantibacillus plantarum* CRL 759 cell-free supernatant	In vitro assay	Agar well diffusion assay was used to determine the antibacterial activity of LP extracts against pathogens isolated from the foot of DFU patients	Restrained development of *P. aeruginosa* and *S. aureus*. Activity attributed to the production of lactic and acetic acid	[140]
*L. bulgaricus, Lactiplantibacillus plantarum*	Topical application for 14 days	Wistar rats induced with diabetes and wounds created	Diabetic wounds accelerated and modulated inflammatory cells	[141]
*Lactococcus chungangensis* CAU 1447 heat-killed lysate	Wound dressing for 7 days	6-week-old male C5BL/6 mice with type 1 diabetes	Reduced wound size and myeloperoxidase (MPO) activity, with early expression of cytokines, growth factors, and chemokines	[142]
*Lactiplantibacillus plantarum* GMNL-6, *Lacticaseibacillus paracasei* GNNL 653 (heat killed)	Topical gels for 20 days	8-week-old female BALB/C mice	Prevented excessive fibrosis, suppression of TGF-beta	[143]
*Lactiplantibacillus plantarum* UBLP 40 (MTCC 5380)	Wound dressing	Infected excision wound model in Lacca mice (wounds created at the back of mice and infected with *S. aureus*)	Effective against *S. aureus* planktonic cells, disrupted biofilm formation, lowered bioburden, and faster wound closure, decreased TNF alpha and LPO levels, increased TGF-beta and antioxidant enzymes	[144]
*Lactiplantibacillus plantarum* ATCC 8014	In vitro and animal studies (48 male SD diabetic rats)	Human umbilical vein endothelial cells (HUVEC)	Healed DFU via regulation of NLRP3 inflammasome	[138]

### 3.5. Animal and human studies investigation

Besides the in vitro and animal model studies, the DFU has also been studied in human subjects. Human studies are essential for validating animal findings and helping in further clinical studies. Majid and colleagues (2016) observed that within 14 days of topical applications of *Lacticaseibacillus casei* and its exopolysaccharide on diabetic wounds (DW) in induced male Wistar diabetic rats, there was a 1.4- and 1.1-fold increase in wound contraction compared to the negative and control groups. Probiotics were also found to stimulate wound healing in diabetic rats [131],[145]. Similar to these studies, another group of researchers evaluated the effect of *Lactiplantibacillus plantarum* gel against burns linked with DW healing in Sprague-Dawley rats. They observed that a topical administration of the strain can enhance healing by lowering inflammation and enhancing hydroxyproline levels, epithelialization, and angiogenesis [146]. Furthermore, Mohtashami et al. (2021) studied the effects of *L. bulgaricus* and *Lactiplantibacillus plantarum* on DW in Wistar rat models. Their findings revealed that the mentioned probiotics could accelerate the healing process of DW and modulate the inflammatory cells in wound sites. Additionally, altered mRNA levels of inflammatory cytokines were observed in wound sites that were treated with the mentioned probiotic strains [141].

Similar to animal studies, a number of studies have evaluated the effects of oral or topical applications of probiotics on human subjects with DFU. Peral et al. (2010) investigated the therapeutic potential of topically applied *Lactiplantibacillus plantarum* against chronic infected foot ulcers in 14 diabetic and 20 non-diabetic patients. According to their results, the wounds were completely healed in diabetic (43%) and non-diabetic patients (50%) only after one month of topical administration of the probiotic strain [127]. In another randomized, double-blind, placebo-controlled trial, the effect of oral probiotic supplementation (daily for 12 weeks) on wound healing and the metabolic status of grade-3 DFU subjects was evaluated. The results showed that oral probiotic supplementation with *Limosilactobacillus fermentum*, *Lacticaseibacillus casei*, *L. acidophilus*, and *B. bifidum* not only lowers the ulcer properties (length, size, and depth) but can also downregulate other metabolic functions like blood glucose and total cholesterol levels [121]. Similar to these studies, Aybar and colleagues (2022), during a 12-week randomized controlled trial, evaluated the effects of topical applications of *Lactiplantibacillus plantarum* ATCC 10241 in DFU subjects receiving surgical wound debridement (SuDe). The outcome of the study showed that SuDe, along with *Lactiplantibacillus plantarum*, could be an effective adjuvant therapy for patients with complicated diabetic foot ulcers [147]. Reinforcing these findings, Moghadam et al. (2022) identified the potential role of *Lactiplantibacillus plantarum* as the topical treatment of second-degree burn wounds in their phase I trials [148].

## 4. Advancement in DFU management: AI-based approach

Most recently, artificial intelligence and machine learning are transforming diabetes mellitus and diabetic foot ulcer management by enabling early detection, diagnosis, and enhanced patient results [149]. Machine learning techniques have emerged as powerful tools for diagnosing and predicting diseases and drug responses [150]. By analyzing huge data sets, the AI algorithms can identify individuals with a high risk of developing DFUs, predict ulcer healing trajectories, and even personalize treatment plans [151]. Researchers demonstrated the promise of ML-based disease diagnosis, which is an inexpensive and time-efficient approach [152]. Multiple ML models have been tailored so as to achieve precision treatment strategies and personalized services for rare diseases [153]. Several studies have used ML techniques for the prediction of diabetes occurrence [154]–[156]. Researchers have meaningfully improved the accuracy of prediction models using ML and AI tools in order to lower the complications of diabetes and enhance patient care [157]. In a recent study, Matboli et al. (2024) utilized comprehensive ML models and predicted type 2 therapeutic targets in a rat model [158]. Similarly, Deberneh et al. (2021) developed an ML-based model that predicts the occurrence of type 2 diabetes [159]. Recently, Rathore et al. (2025) introduced the use of the DFU_XAI framework and found an improvement in the interpretability of deep learning models of DFU labeling and localization, and ensured clinical relevance [168]. Alkhalefah et al. (2025) documented a systematic literature review that explored the use of AI-based tools for prediction, detection, and classification of diabetic foot ulcers [169]. Together, these approaches highlight the importance of AI-based tools in the DFU spectrum.

The microbiome investigation with machine learning models has also shown potential in predicting diabetes. The microbiota of diabetic individuals exhibits disruptions in diversity and abundance that lead to microbial dysbiosis [160]. AI allows us to analyze the gut microbiome compositions of individuals with different genetic profiles and wound characteristics and predict therapeutic strategies for the management of DM and DFU [161]. Most importantly, AI could assist in the formulation of probiotic strains with specific therapeutic applications based on their signature metabolic profiles and functions so as to formulate precision probiotics potentially effective in the treatment and management of DFU [162]. However, several challenges need to be addressed before its full potential can be realized, given its relatively recent emergence.

## 5. Limitations and risk assessment of probiotic therapy

Even though a vast number of in vitro and preclinical studies have elucidated the therapeutic potential of oral supplementations or topical applications of probiotics in DFU patients, several questions remain unanswered. Generally, oral supplements are favored over local applications, as the probiotics in the gut could colonize and improve the gut microbial community, leading to beneficial impacts such as anti-inflammatory, antioxidant, immunomodulatory, and anti-diabetic effects. Conversely, topical applications might offer restricted therapeutic effects, primarily affecting the site of injury, where they decrease the microbial load in the localized area [104]. Additionally, numerous aspects have not yet been covered in the available data. The majority of preclinical studies conducted to date have been either performed on smaller populations and/or are deprived of side effects [163]. Long-term studies are required to cover different aspects, such as identifying novel probiotic strains with enhanced effectiveness, elucidating the effects of the metabolites produced by the probiotic bacteria and their mechanisms of action, appropriate dosage forms, and therapy durations [14].

Additionally, studying possible allergic reactions, complications, and undesirable effects is critical, especially in DFU patients, as well as the possible interactions of probiotics with other dietary resources and anti-diabetic drugs. Currently, most of the research has been conducted on animals, with fewer clinical trials on human subjects. There is a need for randomized controlled trials with the respective probiotic strains, differentiating short-term and long-term effects of probiotics in different DFU individuals. All this could help achieve a definite response regarding the duration of effects of individual probiotic strains or their combination in DFU patients. Collaboration among researchers may potentially enhance the volume of data and its authenticity. Probiotic utilization also produces a small amount of waste, much lower than the other treatment options. However, despite all limitations, probiotics still remain a possible therapeutic solution for treating DFU, given the scarcity of effective medications available for DFU. The diverse range of probiotic strains, each with unique mechanistic actions, offers a valuable solution for addressing this problem [66].

## 6. Future prospectives

The future of probiotics in managing and treating diabetic foot ulcers (DFUs) appears promising, driven by their potential to modulate the gut–skin axis, enhance immune responses, and combat microbial infections. With the increasing number of diabetic populations globally, an increased incidence of chronic non-healing wounds is observed, which has created challenges for their appropriate and effective treatment strategies. The presence of bacterial infections in DFUs could further impede the healing process, leading to chronic wounds. Furthermore, the growing association between multidrug-resistant (MDR) pathogens and diabetic foot ulcers has created challenges for physicians and surgeons in treating DFU without resorting to amputations. Infection with MDR pathogens contributes to increased hospitalization and higher management costs. As antibiotic resistance becomes a growing concern, probiotics offer a safer, adjunctive strategy for promoting wound healing, reducing inflammation, and inhibiting the colonization of pathogenic bacteria and fungi. While probiotics have demonstrated their effectiveness in enhancing wound healing in diabetic patients, and although the role of the microbiome in diabetes has been explored, understanding the association between the microbiome and wound healing remains a challenge and necessitates further research to shed light on these associations.

**Figure 6.**
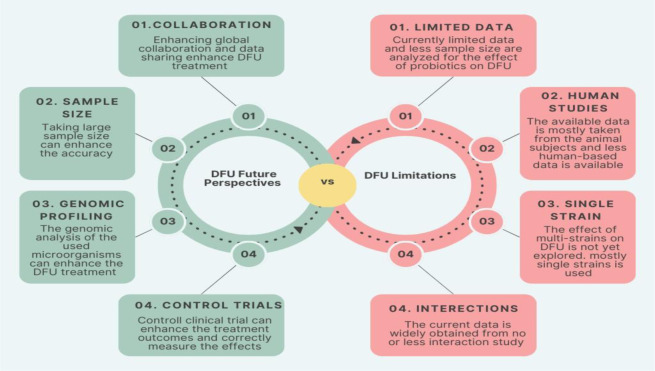
Current limitations of using probiotics in DFU and future recommendations.

Future research is also needed to focus on strain-specific efficacy, delivery systems (topical, oral), and personalized probiotic therapies tailored to individual microbiome profiles. Moreover, integrating probiotics with advanced wound care technologies and biomaterials could further improve therapeutic outcomes. Despite these prospects, large-scale clinical trials and regulatory standardization are essential to establish safety, efficacy, and clinical guidelines for probiotic use in DFU management. The recommendations and future perspectives of probiotics in the context of their DFU treatment and management are summarized in Figure 6. The deployment of these powerful antimicrobial armors in clinical practice is still a challenge and requires extensive clinical research. Many animal-based models have proven the anti-diabetic and wound-healing properties of probiotics. However, in humans, the studies are limited, and some benefits and mechanisms of action of probiotics for the treatment of DFUs have yet to be discovered. The integration of AI presents an exciting new frontier for analyzing robust data on the gut microbiome compositions of individuals with different genetic profiles and wound characteristics. By harnessing the power of AI, novel probiotic interventions could be tailored to the specific needs of each patient, improving wound healing and reducing infection risks in DM patients.

## 7. Conclusions

Diabetic foot ulcers (DFUs) are among the most debilitating complications for individuals with diabetes, and their prevalence continues to rise. Current treatment options for DFUs are limited and often fail to provide effective, long-term healing, leaving many patients to endure prolonged suffering. Probiotics, which are extensively explored for the prevention and treatment of many diseases, can be harnessed for the management and treatment of DFU in patients suffering from this painful condition. They can be used either as a replacement for potentially harmful treatment options that are currently in use or as an adjunct therapy besides other conventional therapeutic strategies. Probiotics possess a range of therapeutic properties that make them promising candidates for the treatment of diabetic foot ulcers. Their ability to restore gut microbiota, inhibit pathogens, deprive nutrients, and produce various postbiotics contributes significantly to their wound-healing potential. These mechanisms collectively support immune modulation, infection control, and tissue regeneration, which are critical for effective DFU management. Existing experimental data, although promising, remains insufficient. Therefore, vigorous validation studies and clinical trials are needed for the implications of probiotics in the treatment of DFUs.
